# Dissecting cancer evolution at the macro-heterogeneity and micro-heterogeneity scale

**DOI:** 10.1016/j.gde.2014.12.001

**Published:** 2015-02

**Authors:** Louise J Barber, Matthew N Davies, Marco Gerlinger

**Affiliations:** 1 Translational Oncogenomics Laboratory, Centre for Evolution and Cancer, Division of Molecular Pathology, The Institute of Cancer Research, 237 Fulham Road, London SW3 6JB, UK; 2 Gastrointestinal Cancer Unit, The Royal Marsden Hospital NHS Foundation Trust, Fulham Road, London SW3 6JJ, UK

## Abstract

Intratumour heterogeneity complicates biomarker discovery and treatment personalization, and pervasive cancer evolution is a key mechanism leading to therapy failure and patient death. Thus, understanding subclonal heterogeneity architectures and cancer evolution processes is critical for the development of effective therapeutic approaches which can control or thwart cancer evolutionary plasticity. Current insights into heterogeneity are mainly limited to the macroheterogeneity level, established by cancer subclones that have undergone significant clonal expansion. Novel single cell sequencing and blood-based subclonal tracking technologies are enabling detailed insights into microheterogeneity and the dynamics of clonal evolution. We assess how this starts to delineate the rules governing cancer evolution and novel angles for more effective therapeutic intervention.

**Current Opinion in Genetics & Development** 2015, **30**:1–6

This review comes from a themed issue on **Cancer genomics**

Edited by **Christine A Iacobuzio-Donahue** and **Elaine A Ostrander**

For a complete overview see the Issue

Available online 31st December 2014

**http://dx.doi.org/10.1016/j.gde.2014.12.001**

0959-437X/© 2014 The Authors. Published by Elsevier Ltd. This is an open access article under the CC BY license (http://creativecommons.org/licenses/by/3.0/).

## Introduction

Cancer is a genetic and epigenetic disease arising from a single cell that has acquired the hallmarks of cancer. Although monoclonal in origin, the background mutation rate and genomic instability mechanisms which are operative in many cancers foster the generation of new mutations during the ensuing expansion of the cancer cell population [1, 2]. Although most new mutations are likely to be deleterious or have no impact on cellular fitness, the enormous number of mutations which can be generated during progression into an advanced cancer, harbouring up to hundreds of billions of malignant cells [3], likely generates a wealth of viable phenotypes. This subclonal diversity is the substrate which Darwinian selection can act upon, permitting the on-going evolutionary adaptation of cancer populations through the expansion of subclones harbouring beneficial aberrations [4].

**Figure 1:**
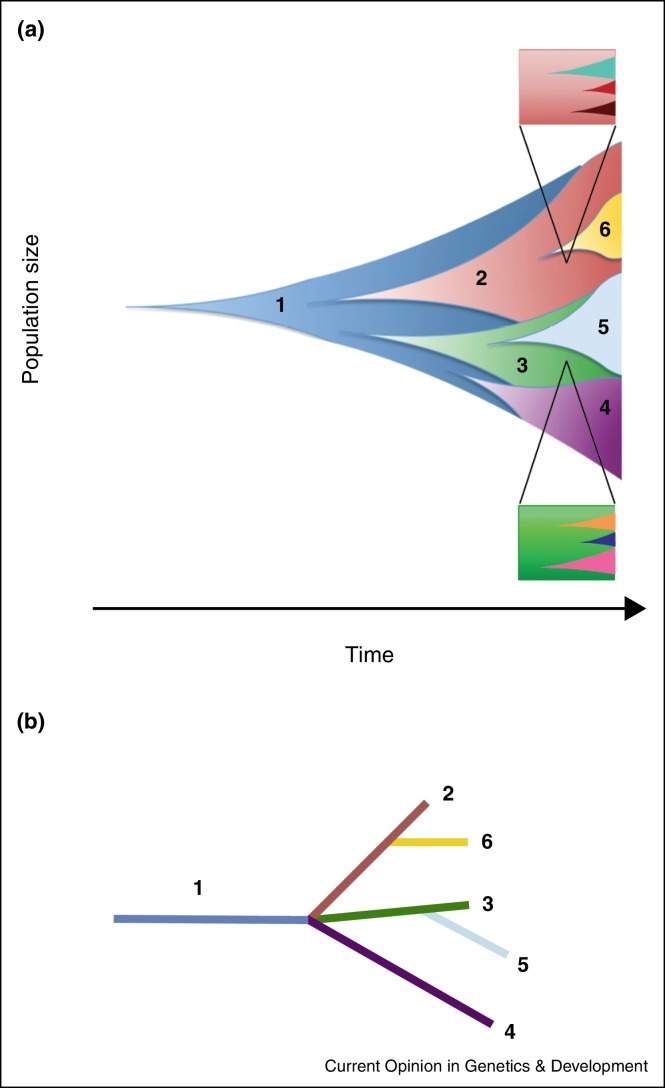
Macro and microheterogeneity in cancer evolution. **(a)** Schematic illustrating clonal evolution. Multiple subclones evolve from the founding clone (blue) and undergo major clonal expansions, changing the composition of the tumour cell population. Subclones are detectable as macroheterogeneity by standard next generation sequencing approaches owing to their large population sizes. Magnifications of small proportions of the cancer cell population (insets) show the population structure at the microheterogeneity level. Newly generated mutations in single cells, which subsequently expand into small subclonal populations are below the detection limit of standard next-generation sequencing techniques and can only be detected through single cell or ultra-deep sequencing technologies. **(b)** Phylogenetic tree reconstructed from the macroheterogeneity data, depicting a branched evolutionary trajectory. The founding clone (blue) represents the trunk of the phylogenetic tree.

However, new mutations, bestowing genetic diversity, are initially confined to individual cells and to small subclones after subsequent rounds of cell division. These remain below the detection limit of standard exome or genome sequencing approaches, which have low sensitivity and high false positive rates when applied for the detection of mutations with allele frequencies below 10% in the DNA extracted from a tumour sample [5]. Thus, this microheterogeneity remains undetectable until a significant expansion of one or more subclones establishes macroheterogeneity (Figure 1a), which may have a branched evolutionary pattern (Figure 1b). Novel single cell [6, 7, 8•] and ultra-deep DNA sequencing technologies [9^•^] only recently started to permit investigations into intratumour micro-heterogeneity *and* macro-heterogeneity, whilst circulating tumour DNA (ctDNA) detection techniques [10, 11, 12, 13] and circulating tumour cell molecular analyses [14] provide insight into the dynamics of evolutionary adaptation. We review how these techniques reveal intratumour micro-heterogeneity and macro-heterogeneity, thereby unravelling the fundamental evolutionary nature of cancer and the central role of genetic intratumour heterogeneity for patient outcome.

## Evidence for intratumour macroheterogeneity

Intratumour macroheterogeneity has been observed across several solid tumour types. Exome sequencing of multiple tumour regions from ten clear cell renal cell carcinomas (ccRCC) demonstrated that, on average, over two thirds of driver somatic copy number aberrations (SCNAs) and of driver mutations were heterogeneous within individual tumours [15^••^]. Subclones were spatially demarcated within primary tumours and differed between primary tumours and metastatic sites within patients. Reconstructing the ancestral relationships of these subclones revealed branched evolutionary patterns with multiple subclones evolving simultaneously in each tumour but along distinct evolutionary paths [15^••^]. A characteristic found in all ten tumours was the presence of inactivating somatic alterations in the von Hippel Lindau (*VHL*) gene and loss of heterozygosity of chromosome 3p, harbouring the second copy of the *VHL* gene, on the trunk of the phylogenetic trees. Thus, these driver aberrations had been acquired early, most likely in the founding cell of each tumour. In contrast, other known ccRCC driver genes, including PI3K-mTOR pathway genes and those encoding epigenetic regulators were predominantly mutated in tumour subclones. *SETD2*, *BAP1* and *PBRM1* driver gene mutations were found in distinct subclones within the same tumour, defying that mutations in these genes define distinct molecular ccRCC subtypes [15^••^]. Studies into signalling pathway activity and prognostic and predictive biomarker expression demonstrated that genetic heterogeneity was associated with phenotypic diversity [15••, 16••, 17]. Multi-region exome sequencing of high-grade serous ovarian cancers also found macroheterogeneity and branched evolution, with early truncal *TP53* mutations in five out of six patients, whereas driver genes such as *PIK3CA*, *CTNNB1* and *NF1* were mutated in subclones [18]. Multi-region SCNA profiling of nine glioblastomas demonstrated homogenous *CDKN2A*/*B* losses and *EGFR* amplifications, suggesting early acquisition on the trunk of the phylogenetic trees [19]. In contrast, SCNAs harbouring *RB1*, *AKT3*, and *MDM4* were always found to be subclonal whereas those affecting *CDK6*, *MET*, *PDGFRA*, *PTEN* and *TP53* were subclonal in some and truncal in other cases [19].

Evidence for macroheterogeneity with significantly expanded subclones has also been identified *within* individual cancer samples. Deep sequencing of triple negative breast cancer biopsies revealed that most *TP53*, *PIK3CA*, and *PTEN* mutations had been acquired early during tumour evolution although they were subclonal in a small proportion of cases [20^•^]. In contrast, mutations in cytoskeletal, cell shape and motility proteins were predominantly subclonal, suggesting on-going evolutionary adaptation. Fluorescence in situ hybridisation of driver SCNAs identified genetically distinct subclonal populations in the majority of *ETV6-RUNX1* positive acute lymphoblastic leukaemias (ALL) [21]. Twenty-four cases exhibited branched evolution and only six malignancies followed a linear evolutionary pattern [21]. Sequencing of single biopsies from non-small cell lung cancers (NSCLC) revealed subclonal heterogeneity in ten out of 17 cases [22]. Tumours harbouring *KRAS* or *EGFR* mutations had always acquired these in the founding clone, whereas putative driver mutations in *HGF* were subclonal. A large study investigating mutation concordance within NSCLC primary tumours and between primary tumours and metastases or recurrences found no macroheterogeneity of *EGFR* driver mutations, further supporting the notion that activating *EGFR* mutations are generally truncal [23]. This was also confirmed by two recent NSCLC multi-region exome sequencing studies. All identified activating *EGFR* mutations and indeed the majority of all other known NSCLC driver mutations and driver SCNAs were located on the trunks of the phylogenetic trees [24, 25]. Macroheterogeneity and branched phylogenetic patterns were nevertheless identified in each tumour. Although most heterogeneous aberrations may be passengers, the high mutation rate in NSCLCs impairs the ability to define the driver gene catalogue of these tumours [26] and subclonal drivers may have remained undetected as a consequence. Mutational signature analysis showed that a cell-endogenous mutational process caused by up-regulation of the APOBEC deaminase [24, 25] was the predominant mechanism of NSCLC subclonal mutation generation [27^••^], even in patients with on-going tobacco smoke exposure.

## Insights into intratumour microheterogeneity

Novel sequencing technologies increasingly allow the investigation of microheterogeneity at the fundamental level of the single cell. The detection of SCNAs and point mutations in up to 60 individual cell nuclei from each of two breast cancers identified major subclones evolving in a branched evolutionary fashion in each tumour [8^•^], corroborating the conclusion from breast cancer macroheterogeneity studies [20^•^]. Single cell resolution further revealed relatively stable SCNA profiles across cancer cells within a tumour whereas point mutations differed between major subclonal populations but also within subclones. Thus, SCNAs had been acquired early during carcinogenesis and point mutation acquisition was continuously driving microheterogeneity generation. Mutation rate estimates based on this data revealed ∼8 new mutations per cell division in a triple negative cancer and 0.6–0.9 new mutations in an ER positive tumour, which is similar to the estimated 0.6 new mutations per division for normal cells. Importantly, single cell mutational heterogeneity allows insights into current mutation rates. In contrast, mutations observed at the macroheterogeneity level have been acquired many generations before clonal expansion made them detectable and only provides a historical record of the mutational processes that were operative in earlier tumour stages [28^•^].

Reconstruction of SCNA profiles from single cell RNA sequencing data from glioblastomas identified a monoclonal structure in four cases and two major subclones within one further case [29^••^]. Within the limits of the assay, which has a low sensitivity to detect small aberrations, SCNAs were similar between individual cells of a clone or subclone. Thus, the generation of new SCNAs may be a rare event and the observed profiles were likely acquired early during cancer evolution, similar to the results in breast cancers [8^•^]. Single cell RNA expression data further enabled the simultaneous assessment of gene expression profiles between single cells with similar SCNA profiles. This detected transcriptional signatures of different glioblastoma subtypes and variable degrees of stemness co-existing in different cells within a tumour. The simultaneous interrogation of genetic and non-genetic macroheterogeneity within a cancer cell population provides powerful opportunities to assess phenotypic consequences of subclonal genetic aberrations.

Macroheterogeneity of known driver mutations is rare between primary colorectal cancers (CRCs) and associated metastatic lesions. Mutations in *KRAS*, *NRAS*, *BRAF* and *APC* driver genes were always concordant and only low-level discordance was observed for mutations in *TP53*, *PIK3CA* and *PTEN* in a study of 69 primary CRC and metastasis pairs [30]. The absence of macroheterogeneity, for example for *KRAS* and *NRAS* mutations, suggests that these drivers were acquired on the trunk of the phylogenetic tree, in tumours in which they are detectable. The high concordance most likely explains the robust performance of *KRAS* and *NRAS* mutations as predictors of primary resistance to anti-EGFR therapy in CRCs [31, 32]. However, *KRAS and NRAS* mutations became detectable in the ctDNA from 23 out of 24 initially *KRAS/NRAS* wild-type CRCs at the time acquired resistance to anti-EGFR treatment had developed [33^••^]. Surprisingly, multiple distinct activating *KRAS* and *NRAS* mutations emerged in the ctDNA in 63% of patients, demonstrating that polyclonal resistance evolution was common. An analysis of the kinetics of *KRAS* mutation evolution in these patients further concluded that *KRAS* mutations had been present before anti-EGFR therapy initiation, in small subclones comprising ∼2000–3000 cancer cells [34]. Direct support for this microheterogeneity has been provided by the detection of low level *KRAS* mutations by sensitive digital PCR technology in patients found to be *KRAS* wild-type by standard detection techniques [41]. Thus, microheterogeneity of *KRAS* mutations and potentially also of other resistance driver mutations is likely to be present in many metastatic CRCs which are *KRAS* wild-type based on standard sequencing approaches. These mutations may evade detection owing to the small number of affected cells and through spatial segregation across metastatic sites but they eventually drive resistance evolution and therapy failure. The reliable evolution of one or multiple *KRAS* or *NRAS* mutant subclones in most patients during anti-EGFR therapy further suggest that the population size and mutation rates are sufficiently high to generate many beneficial driver mutations in any metastatic CRC. The presence of *KRAS* mutation microheterogeneity in many tumours which are *KRAS* wild-type by standard sequencing technologies together with the absence of *KRAS* mutational macroheterogeneity further indicated that these new subclones rarely undergo significant clonal expansion. Thus, *KRAS* mutations apparently have a low or no selective advantage unless they are acquired early during carcinogenesis or the tumour is treated with EGFR-targeted agents.

**Figure 2:**
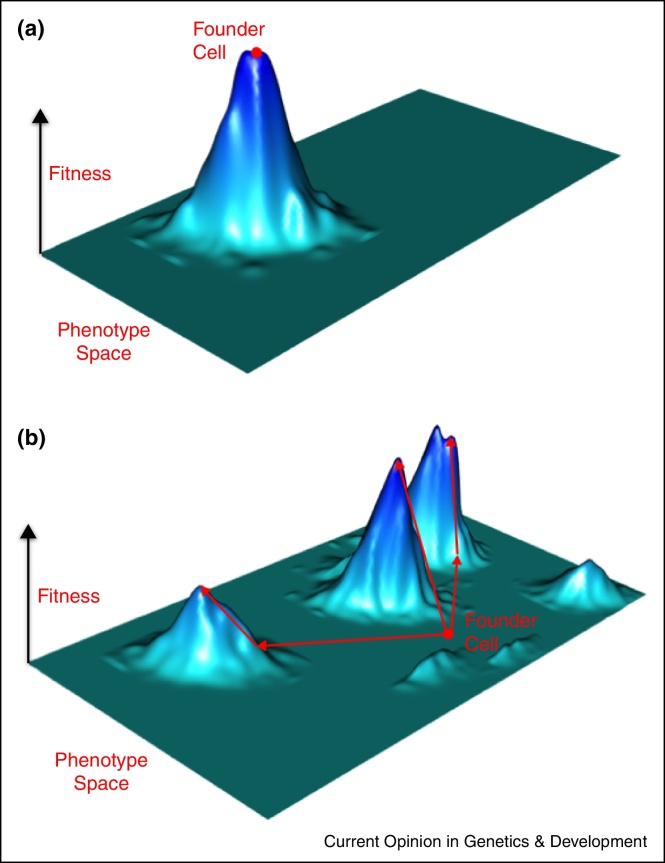
Influence of the fitness landscape on cancer evolutionary patterns. **(a)** Hypothetical cancer fitness landscape in which the founding cell (red dot) is already located at a fitness peak. Further evolutionary adaptation is only possible through a change in the fitness landscape, for example through a change in the environment or through drug therapy. Microheterogeneity can be extensive in this tumour but macroheterogeneity is absent. **(b)** Hypothetical fitness landscape where the founding cell is not located at a fitness peak. Tumour subclones can increase their relative fitness through the acquisition of further driver mutations which will lead to subclonal expansion. Increases in fitness are illustrated as arrows climbing up the fitness peaks. If multiple subclones acquire drivers that increase their relative fitness, branched evolution can occur. Multiple fitness peaks indicate multiple possible phenotypes which lead to increased cellular fitness.

The presence of strong driver aberrations in the founding cell of most CRCs which leaves only limited opportunity to further optimize cancer cell fitness could be a parsimonious explanation for this paradox. In other words, many CRCs may already occupy a fitness peak on the fitness landscape at the time of cancer initiation (Figure 2a) precluding significant expansion and macroheterogeneity evolution of subclones harbouring additional drivers. This may be fundamentally different in the founding cell of a ccRCC (Figure 2b), which may only harbour a small number of weak drivers such as mutations in *VHL* and chromosome 3p loss. This is supported by studies in patients with germ-line *VHL* mutations which only showed a modest proliferative advantage of biallelic *VHL* inactivation in renal tubular cells [35] compared to the proliferative advantage conferred by biallelic inactivation of the *APC* tumour suppressor gene (which is altered in ∼80% of CRCs [36]) in colon cells. Thus, the founder clone of a typical ccRCC is likely to be located on a fitness landscape that permits significant further fitness increments through the acquisition of additional driver aberrations (Figure 2b). This would explain the frequent evolution of subclones harbouring additional driver genes and the detection of macroheterogeneity in these tumours.

## Conclusions

Exome and genome sequencing studies of up to 500 cancer samples recently identified the most prevalent driver genes in many cancer types. In parallel, smaller studies started to portray the subclonal landscapes of many tumour types at the macroheterogeneity level through multi-region sequencing approaches or subclonal composition analysis of individual biopsies. This provided ample evidence for on-going evolutionary adaptation during cancer progression, frequently along complex branched trajectories, and started to delineate the spatial structures of subclonal architectures. The spatial segregation of functionally distinct subclones is a major hurdle for personalized cancer therapies as it complicates efforts to accurately assess the driver aberration landscapes of individual tumours. These results also question whether and how tumours harbouring subclones with different driver mutations can be optimally treated. The concept of a clinically dominant clone, which is not necessarily numerically dominant in a cancer but ultimately lethal for an individual patient [37•, 38], is emerging from this work and the development of strategies to detect, track and treat clinically dominant subclones is an important area of future research. At the same time, macroheterogeneity studies started to define cancer type specific ‘evolutionary rules’, such as the identification of driver genes which are commonly altered on the trunk of a specific tumour type, providing opportunities to prioritize the development of targeted therapeutics [39]. Most recently, new technologies enabled the study of microheterogeneity in exceedingly small subclones and even at the quantum level of the single cell. Combined with assessments of subclonal population dynamics through ctDNA or circulating tumour cell tracking [14], these tools start to unravel key mechanisms of cancer evolution at an unprecedented level of detail. For example, quantification of de novo mutation generation, the construction of genotype-phenotype maps and ultimately the mapping of dynamic fitness landscapes can now be accomplished. As on-going cancer evolution fosters cancer progression and therapy failure [40], a fundamental understanding of the rules governing cancer evolution may lead to novel therapeutic and preventive approaches to slow down or thwart evolution in order to improve clinical outcomes.

## References and recommended reading

Papers of particular interest, published within the period of review, have been highlighted as:

• of special interest
•• of outstanding interest
